# Hypertension in India: a gender-based study of prevalence and associated risk factors

**DOI:** 10.1186/s12889-024-20097-5

**Published:** 2024-10-01

**Authors:** Raza Mohammad, Dhananjay W. Bansod

**Affiliations:** 1 International Institute for Population Sciences, Mumbai, 400088 India; 2 Department of Public Health and Mortality Studies, International Institute for Population Sciences, Mumbai, 400088 India

**Keywords:** Hypertension, Risk factors, NFHS, Age, Gender, India

## Abstract

**Background:**

Hypertension, a major non-communicable disease, is responsible for a significant number of global deaths, including approximately 17.9 million yearly. The Global Burden of Disease 2019 (GBD 2019) estimates that 19% of global deaths are attributed to elevated blood pressure. India, with a population of over 1.4 billion, is facing a serious challenge in combating this silent killer. This study aims to analyze the gender-based prevalence of hypertension in India and explore its associated risk factors using data from the fifth National Family Health Survey (NFHS-5).

**Methods:**

NFHS-5 collected data from 636,699 households across all states and union territories. The study includes standardized blood pressure measurements for 17,08,241 individuals aged 15 and above. The data were analyzed using *Stata*, employing descriptive statistics for the assessment of the prevalence and binary logistic regression to identify predictors of hypertension.

**Results:**

The study found the overall prevalence of hypertension in India to be 22.6%, with men (24.1%) having a higher prevalence than women (21.2%). Prevalence increased with age, reaching 48.4% in individuals aged 60 and above. Urban residents had a slightly higher prevalence (25%) than rural residents (21.4%), indicating the rapid spread of hypertension across all populations. Regional variations were observed, with the highest prevalence in Sikkim (37.9%) and the lowest in Rajasthan (16.5%). Increasing age, urban residence, belonging to certain religions (Muslim and other than Hindu or Muslim), and being classified as Scheduled Tribes (ST) were associated with a higher likelihood of hypertension. Conversely, belonging to Scheduled Castes (SC) or Other Backward Classes (OBC), being currently unmarried, and having higher education were associated with a lower likelihood of hypertension. Wealth index analysis revealed that those in the richest quintile were more likely to have hypertension. Behavioural risk factors, such as alcohol consumption, overweight, obesity, increased waist circumference, and high blood glucose levels, are positively associated with hypertension.

**Conclusion:**

Hypertension is a significant health burden in India, affecting both men and women. Age is the strongest non-modifiable predictor for both men and women. However, ageing women have higher odds of hypertension than ageing men, and this distinction becomes much more evident in their older ages. Obese women, based on BMI, have higher odds of hypertension than men. However, hypertension prevalence is slightly higher among men who are overweight or obese compared to women. BMI, waist circumference, random glucose level, alcohol use, and education level emerged as major predictors. Health education and awareness campaigns are critical to control the growing burden of hypertension in India. Policymakers must focus on preventive measures, targeting lifestyle changes and improved healthcare access for both men and women. By addressing these risk factors, India can make significant progress in controlling hypertension and reducing its impact on public health.

**Supplementary Information:**

The online version contains supplementary material available at 10.1186/s12889-024-20097-5.

## Background

Non-communicable diseases account for 41 million deaths every year, equivalent to 74% of all deaths globally. Cardiovascular disease accounts for approximately 17.9 million deaths yearly, nearly one-third of the total deaths [1]. According to the Global Burden of Disease 2019 (GBD 2019) estimates, the leading metabolic risk factor globally is elevated blood pressure, to which 19% of global deaths are attributed [2].

Hypertension is a major public health problem due to its high prevalence worldwide [3–5]. As per World Health Organization (WHO), The number of adults with hypertension increased from 594 million in 1975 to 1.13 billion in 2015, with the increase seen mainly in low- and middle-income countries [6].

Hypertension is a silent killer as very rarely can any symptom be seen in its early stages until a severe medical crisis occurs, like heart attack, stroke, or chronic kidney disease [6, 7]. The only way to detect hypertension is to have a health professional measure blood pressure. Having blood pressure measured is quick and painless. Although individuals can measure their blood pressure using automated devices, an evaluation by a health professional is important for assessing risk and associated conditions [8].

Hypertension or high blood pressure (BP) is abnormally high arterial blood pressure. Since people are unaware of excessive blood pressure, it is only through measurements that detection can be done. Although most patients with hypertension remain asymptomatic, some patients report headaches, light-headedness, vertigo, altered vision, or fainting episodes [9].

The WHO is supporting countries in reducing hypertension as a public health problem. In 2021, the WHO released a new guideline for the pharmacological treatment of hypertension in adults [8, 10]. The publication provides evidence-based recommendations for the initiation of treatment of hypertension and recommended intervals for follow-up. The document also includes target blood pressure to be achieved for control and information on who can initiate treatment in the healthcare system [10].

To support governments in strengthening the prevention and control of cardiovascular disease, WHO and the United States Centers for Disease Control and Prevention (U.S. CDC) launched the Global Hearts Initiative, which includes the HEARTS technical package. The six modules of the HEARTS technical package (Healthy-lifestyle counselling, Evidence-based treatment protocols, Access to essential medicines and technology, Risk-based management, Team-based care, and Systems for monitoring) provide a strategic approach to improve cardiovascular health in countries across the world [11].

In September 2016, WHO began a partnership with Resolve to Save Lives, an initiative of Vital Strategies, to support national governments in implementing the Global Hearts Initiative. Since implementing the programme in 18 low- and middle-income countries, 3 million people have been put on protocol-based hypertension treatment through person-centred models of care. These programmes demonstrate the feasibility and effectiveness of standardized hypertension control programmes [10].

Hypertension prevalence would increase to 44% (CI: 43–45) in year 2030, a relative increase of 17%, instead of a relative decline of 25% by the year 2030 as proposed by the WHO [12].

Modifiable risk factors for hypertension include unhealthy diets (excessive salt consumption, a diet high in saturated fat and trans fats, low intake of fruits and vegetables), physical inactivity, consumption of tobacco and alcohol, being overweight or obese, increased waist circumference and having high glucose levels. Non-modifiable risk factors include a family history of hypertension, age over 65 years and co-existing diseases such as diabetes or kidney disease [6].

According to a community-based cross-sectional study carried out by the National Nutrition Monitoring Bureau (NNMB) in 120 Integrated Tribal Development Authority (ITDA) villages in nine States viz. Andhra Pradesh, Gujarat, Karnataka, Kerala, Maharashtra, Madhya Pradesh, Odisha, Tamil Nadu and West Bengal during 2007–2008. The prevalence of hypertension was 27.1 and 26.4% among men and women, respectively. It was higher in the states of Odisha (50-54.4%) and Kerala (36.7–45%) and lowest in Gujarat (7-11.5%) [13].

A study was conducted in the year 2014-15 in three regions in rural India: Trivandrum (Kerala), (West) Godavari (Andhra Pradesh) and the Rishi Valley (Andhra Pradesh) found that 47.7% overweight persons and 39.6% higher waist circumference were hypertensive [14]. Another community-based cross-sectional study found that hypertension prevalence was high in West Bengal (29.5%) and Kerala (28.9%) and low in Madhya Pradesh (16%) and Uttar Pradesh (19%) [15].

The prevalence of hypertension in India in 2015-16 was 11.3% among persons aged between 15 and 49 and was 4% points higher among men than among women. Persons in the urban location (12.5%) had a marginally higher prevalence than persons in rural location (10.6%). The proportion of population suffering from hypertension varied greatly between states, with a prevalence of 8.2% in Kerala to 20.3% in Sikkim [16].

The risk of hypertension was 6–8 times higher in elderly people and 2–3 times in 35–59 years compared with 20–34 years. Men with overweight and obesity were at 1.69 (CI: 1.43–2.01) and 2.42 (CI: 2.01–2.91) times higher risk of hypertension, respectively, while it was 2.03 (CI: 1.77–2.33) and 2.35 (CI: 2.12–2.60) times higher in women [13]. Those using tobacco and consuming alcohol were at a higher risk of hypertension compared with the non-users. Advancing age, obesity/overweight, sex, socioeconomic status and consumption of alcohol were found to be the major predictors of hypertension [13, 16, 17].

Hypertension is a major cause of premature death worldwide [8]. Raised blood pressure (BP) is a significant risk factor for chronic heart disease, stroke, and coronary heart disease. Elevated BP is positively correlated to stroke and coronary heart disease risk. Other than coronary heart disease and stroke, its complications include heart failure, peripheral vascular disease, renal impairment, retinal haemorrhage, and visual impairment [6]. One of the global targets for non-communicable diseases is reducing hypertension prevalence by 33% between 2010 and 2030 [18].

While the existing literature has valuable information on the risk factors associated with hypertension [6, 13–17], there is a need for a comprehensive gender-based analysis, which is critical for understanding the diverse risk factors and modifying interventions for both genders accordingly. The gender-specific dynamics for hypertension are crucial to investigate the prevalence and associated risk factors. Hence, the present study intends to provide rigorous information on the gender-based prevalence of hypertension among persons aged 15 and above and its associated risk factors in India.

## Methods

The present study uses the most recent Demographic and Health Survey (DHS) in India, i.e., the fifth National Family Health Survey (NFHS-5) round. The NFHS is an Indian version of the DHS that provides consistent and reliable estimates of fertility, mortality, family planning, utilisation of maternal and child health care services and other related indicators at the national, state and regional levels. NFHS surveys have been conducted under the stewardship of the Ministry of Health and Family Welfare (MoHFW), Government of India. MoHFW designated the International Institute for Population Sciences (IIPS), Mumbai, as the survey’s nodal agency. Inner City Fund (ICF) provided technical assistance through the DHS Program, which is funded by USAID.

The International Institute for Population Sciences coordinated each round of the survey with support from several international organizations. The sampling, questionnaire structure, and content of the NFHS surveys follow what has been adopted by the Demographic Health Surveys (DHS) in other developing countries. The NFHS uses nationally representative area-based sampling frames in each survey.

NFHS adopted a two-stage stratified random sampling approach by selecting primary sampling units (PSUs) (villages in rural areas and census enumeration blocks in urban areas) with probability proportional to population size (PPS) at the first stage and subsequently, picking the same number of households from each of selected PSUs through systematic random sampling. Both male and female interviewers were recruited by field agencies to interview respondents of the same sex. The data collection team made up to three visits in case nobody was present in the chosen household or any eligible member was not available at the time of the household visit.

The NFHS-5 was conducted in two phases (phase I from 17 June 2019 to 30 January 2020 and phase II from 2 January 2020 to 30 April 2021). The survey was conducted across all 28 states and 8 union territories (UTs) in India. The survey is representative not only at the national and state level but also at the district level. NFHS-5 provides information from 707 districts of India, which gathered data from 636,699 households across all 28 states and 8 union territories (UTs) in India.

For the first time, NFHS-5 has expanded the age range for the measurement of blood pressure. Blood pressure was measured for all women and men aged 15 and above using *an Omron Blood Pressure Monitor* to determine the prevalence of hypertension. Blood Pressure measurements for each respondent were taken three times with an interval of five minutes between readings. Respondents whose average systolic blood pressure (SBP) was more than 130 mm Hg and/or whose average diastolic blood pressure (DBP) was more than 85 mm Hg were considered to have elevated blood pressure readings, and they were encouraged to see a doctor for a full evaluation.

The response rate for BP measurements was 91.3% among women and 81.8% among men. Apart from taking BP measurements, all participants, irrespective of their BP, were asked, ‘Were you told on two or more different occasions by a doctor or other health professional that you had hypertension or high blood pressure?’ If they responded in the affirmative, they faced a follow-on question, ‘To lower your blood pressure, are you taking a prescribed medicine?’ and recorded [19].

The analysis was performed on women and men aged 15 and 95 + years from the Person Recode: IAPR7DDT. ZIP file accessed from the Demography and Health Survey (DHS); hypertension was considered the outcome variable for the present study. Three blood pressure readings were taken in NFHS with an interval of five minutes between readings. The first measurement was discarded, and based on the average of the second and third blood pressure readings, whether a participant is hypertensive has been decided [16].

### Dependent variable

Systolic blood pressure (SBP) is the degree of force when the heart is pumping (contracting), and Diastolic blood pressure (DBP) is the degree of force when the heart is relaxed.

The definition based on the criteria given by WHO and the American Heart Association, an individual is classified as having hypertension if they have a systolic blood pressure (SBP) level greater than or equal to 140 mmHg (millimetres of mercury) or diastolic blood pressure (DBP) greater than or equal to 90 mmHg (millimetres of mercury). In addition, an individual is classified as having hypertension if they are taking anti-hypertensive medication to lower their blood pressure [20].

Hypertension for persons aged between 15 and over. The dichotomous variable, hypertension, has been defined as 1 = hypertensive, else = 0.

### Explanatory variables

Predictors have been selected based on their effects on hypertension. The sociodemographic variables used are age, sex, place of residence, region of residence, religion of the household head, caste, current marital status, education level, and wealth index. Besides sociodemographic variables, tobacco use and alcohol consumption, body mass index (BMI), waist circumference, and random glucose level are used as proxies for behavioural risk factors.

The country has a wide geographical distribution, and for the purpose of the study, it is divided into six regions – North (Jammu & Kashmir, Himachal Pradesh, Punjab, Chandigarh, Uttarakhand, Haryana, NCT of Delhi, Rajasthan, and Ladakh), Central (Uttar Pradesh, Chhattisgarh, and Madhya Pradesh), East (Bihar, West Bengal, Jharkhand, and Odisha), Northeast (Sikkim, Arunachal Pradesh, Nagaland, Manipur, Mizoram, Tripura, Meghalaya, and Assam), West (Gujarat, Dadra & Nagar Haveli and Daman & Diu, Maharashtra, and Goa), and South (Andhra Pradesh, Karnataka, Lakshadweep, Kerala, Tamil Nadu, Puducherry, Andaman & Nicobar Islands, and Telangana).

The person who does not belong to any caste or tribe or has chosen not to respond has been categorised as ‘None of them’ in the caste or Tribe variable. All the people who are not currently married, such as a person who never married, or a widow, a widower, divorced or separated, have been categorised as Others.

The wealth index is a proxy indicator for socioeconomic scale based on scores on ownership of consumer goods and household characteristics, such as availability of basic facilities like clean drinking water, owning of televisions and non‑motor two-wheelers, type of housing, drinking water access and facilities for sanitation. All households are classified into wealth quintiles ranging from Richest to Poorest, with in-between classes as Richer, Middle and Poorer.

The BMI categories are defined as Thin < 18.5 (kg/m^2^), Normal 18.5–24.9 (kg/m^2^), Overweight 25.0-29.9 (kg/m^2^) and Obese ≥ 30.0 (kg/m^2^). The NFHS-5 collected anthropometric data on the height and weight of women aged 15–49 and men aged 15–54, so the person above the prescribed age, women who are pregnant or have given birth in the last two months, were categorised as BMI not taken [19].

The information on waist circumference is also, for the first time in NFHS-5, measured for women and men aged 15–49 years. The waist circumference is Normal when it is less than 80 cm (centimetre) for women and 94 cm for men. It is categorised as the ‘increased risk of metabolic complications’ when more than 80 cm and 94 cm for women and men, respectively. The persons above the prescribed age, those who did not participate, pregnant women and women who had a birth in the preceding two months were categorised as ‘Not measured’.

In NFHS-5, the age range for random blood glucose has been extended to 15 and above. Random blood glucose was the measurement of blood glucose at any time without the necessity of fasting. It is classified as ‘High’ when found to be more than 140 mg/dl (milligrams per deciliter) for both men and women and ‘Normal’ when it is less than 140 mg/dl. The person who refused to participate, not tested or other in blood glucose measurement is categorised as ‘Not measured’ [19].

### Statistical analysis

All statistical analyses were performed using Stata SE *version 17.0* (Stata Corporation. 2021. Stata: Release 17, College Station, Texas). The overall weighted prevalence of hypertension has been reported based on background characteristics. Also, the weighted hypertension prevalence has been calculated for both men and women.

Univariate and multivariate binary logistic regression analyses have been used to understand the prevalence of hypertension and determine the associated factors. Missing values were excluded from the analysis. Also, the appropriate weight has been used. The model for logistics regression is given below,

$$\begin{gathered}{\text{lo}}{{\text{g}}_{\text{e}}}{\text{[P(}}{{\text{Y}}_{\text{i}}}{\text{ = 1|}}{{\text{X}}_{\text{i}}}{\text{)/1 - P(}}{{\text{Y}}_{\text{i}}}{\text{ = 1|}}{{\text{X}}_{\text{i}}}{\text{)]}} \hfill \\{\text{ = lo}}{{\text{g}}_{\text{e}}}\left[ {{{\pi /1 - \pi }}} \right]{{ = \alpha + }}{{{\beta }}_{\text{1}}}{{\text{X}}_{{\text{i1}}}}{\text{,}}{{{\beta }}_{\text{k}}}{{\text{X}}_{{\text{ik}}}} \hfill \\ \end{gathered}$$

Where, Yi is the binary response variable, and Xi is the set of explanatory variables like socioeconomic characteristics and risk factors. The dependent variable for the study is the overall prevalence of hypertension. Also, hypertension prevalence among men and women is used separately as a dependent variable.

Variables significant in the unadjusted analysis at *p* < 0.05 are considered for incorporation into the multivariable analysis. Multicollinearity has been assessed by variance inflation factors (VIF); explanatory variables with VIF greater than or equal to 10 are considered for removal from the multivariable model. However, no multicollinearity has been observed throughout the analysis. Crude odds ratios (CORs) and adjusted odds ratios (AORs), along with the sample size used, are reported separately for men, women and overall people.

## Results

The prevalence of hypertension is found to be 22.6% in India (24.1% in men and 21.2% in women) from 17,08,241 persons [Men: 7,85,611 (46%), Women: 9,22,630 (54%)] who participated in the survey (Supplementary Table 1a). This indicates that one in every fifth individual is found hypertensive in India. (Table 1)

**Table 1:** Prevalence of hypertension among men and women age 15 and over, according to background characteristics, India, 2019-21

Background characteristics	Men				Women				Overall		
	Prevalence of hypertension (%)		Weighted sample		Prevalence of hypertension (%)		Weighted sample		Prevalence of hypertension (%)		Weighted sample
**Total**	**24.1**		**7**,**85**,**611**		**21.2**		**9**,**22**,**630**		**22.6**		**17**,**08**,**241**
**Age of Household Member**											
15–29	8.3		2,63,624		4.8		3,25,911		6.4		5,89,534
30–44	21.6		2,12,674		15.4		2,55,549		18.2		4,68,223
45–59	34.2		1,69,044		34.1		1,99,619		34.2		3,68,663
60 and over	45.5		1,40,188		51.3		1,41,410		48.4		2,81,598
**Type of Place of Residence**											
Urban	26.7		2,52,345		23.4		2,89,463		25.0		5,41,808
Rural	22.9		5,33,266		20.2		6,33,168		21.4		11,66,433
**Region of Residence**											
North	24.8		1,07,673		20.6		1,26,859		22.5		2,34,533
Central	22.6		1,90,448		19.3		2,24,242		20.8		4,14,690
East	20.7		1,66,058		18.6		2,05,101		19.5		3,71,160
Northeast	23.0		26,791		20.4		30,061		21.6		56,852
West	22.6		1,25,109		21.9		1,34,099		22.3		2,59,207
South	30.0		1,69,531		26.1		2,02,268		27.9		3,71,799
**Religion of The Household Head**											
Hindu	24.0		6,54,793		20.9		7,62,342		22.4		14,17,134
Muslim	21.6		88,190		20.9		1,10,402		21.2		1,98,592
Others	30.4		42,627		26.6		49,887		28.4		92,515
**Caste/Tribe of The Household Head**											
None of them^#^	26.2		2,04,388		23.8		2,39,551		24.9		4,43,940
Schedule Caste (SC)	22.9		1,70,280		19.4		2,01,453		21.0		3,71,732
Schedule Tribe (ST)	22.3		75,903		19.6		86,936		20.9		1,62,838
Other Backward Classes (OBC)	23.9		3,35,040		21.0		3,94,691		22.3		7,29,731
**Current Marital Status**											
Married	29.3		5,43,247		20.9		6,48,239		24.7		11,91,486
Unmarried	12.6		2,42,282		21.9		2,74,250		17.6		5,16,532
**Highest Educational Level Attained**											
Non-literate	29.3		1,27,157		30.9		3,09,481		30.4		4,36,638
Primary	28.7		1,13,329		26.3		1,26,143		27.4		2,39,472
Secondary	21.9		4,13,798		14.5		3,79,091		18.4		7,92,889
Higher	22.0		1,30,726		10.9		1,07,557		17.0		2,38,283
**Wealth Index**											
Poorest	19.6		1,40,038		18.5		1,74,228		19.0		3,14,266
Poorer	20.9		1,53,985		19.1		1,83,719		19.9		3,37,704
Middle	23.3		1,64,343		21.1		1,88,752		22.1		3,53,095
Richer	26.2		1,66,409		22.5		1,90,920		24.2		3,57,329
Richest	29.8		1,60,836		24.8		1,85,012		27.1		3,45,847
**Smokes or Uses Tobacco**											
No	22.4		4,76,082		20.3		8,38,898		21.1		13,14,981
Yes	26.8		3,08,509		30.7		83,616		27.6		3,92,125
**Drinks Alcohol**											
No	22.7		6,32,474		21.1		9,10,500		21.7		15,42,973
Yes	30.2		1,52,011		30.7		12,024		30.2		1,64,035
**Body Mass Index (BMI in kg/m** ^**2**^ **)**											
Normal (BMI 18.5–24.9)	15.9		54,684		9.4		3,79,292		10.3		4,33,976
Thin (BMI < 18.5)	8.0		13,911		5.9		1,21,729		6.1		1,35,640
Overweight (BMI 25.0-29.9)	30.3		16,957		19.2		1,14,894		20.6		1,31,851
Obese (BMI ≥ 30.0)	39.4		3,571		27.9		41,291		28.8		44,862
BMI not measured	24.9		6,96,488		44.9		2,65,424		30.4		9,61,912
**Waist Circumference**											
Normal (women: (≤ 80 cm); men (≤ 94 cm))	15.6		78,375		7.6		3,91,157		8.9		4,69,532
Increased risk of metabolic complications (women: (> 80 cm); men (> 94 cm))	38.5		10,851		17.6		2,66,096		18.4		2,76,947
Not measured	24.9		6,96,384		44.9		2,65,378		30.4		9,61,762
**Random Blood Glucose Level**											
Normal (≤ 140 mg/dl)	20.0		6,56,638		17.2		7,88,905		18.5		14,45,543
High (> 140 mg/dl)	40.3		1,11,570		40.1		1,11,794		40.2		2,23,364
Not measured	78.6		17,402		69.4		21,932		73.3		39,334

*Note*^#^ Including those who do not belong to any caste/tribe, or have chosen not to respond

The results indicate that the prevalence of hypertension increases as age increases (6.4% in 15–29 aged to 48.4% in those aged 60 and over). The prevalence is slightly higher in men compared to women between the ages of 15 and 59. Nevertheless, in older ages 60 and over, hypertension prevalence is about 6% higher in women (51.3%) than in men (45.5%).

Hypertension prevalence is 25% in urban areas, compared with 21.4% in rural areas. 26.7% in urban men compared to 22.9% in rural men. Also, in women 23.4% in urban compared to 20.2% in rural women.

The prevalence of hypertension varies across regions. The lowest is found in the Eastern region (19.5%), whereas the highest prevalence is found in the Southern region (27.9%). The prevalence is found to be slightly higher in men compared to women in all the regions.

The prevalence of hypertension among Hindus is 22.4%, whereas it is 21.2% in Muslims. It is indicated that hypertension prevalence is higher (28.4%) among people belonging to other than the Hindu or Muslim religion. Hypertension prevalence is less in persons belonging to Schedule Tribe (20.9%) and high in persons with no caste or tribe (24.9%).

The prevalence of hypertension is 24.7% in the currently married person compared with the person whose marital status is others (17.6%). It is also indicated that married men are more hypertensive (29.3%) than women (20.9%). However, among those whose marital status is others, hypertension is more prevalent in women (21.9%) than in men (12.6%).

The hypertension prevalence is found to be more in non-literates (30.4%) and less in people with a higher level of education (17.0%). Also, it describes that non-literate women have higher hypertension (30.9%) than men (29.3%), whereas women with a higher level of education have lesser (10.9%) hypertension than men (22.0%).

Nevertheless, persons from the poorest wealth index are less (19.0%) hypertensive than persons from the richest (27.1%). Hypertension increases as the wealth index increases.

Hypertension is more prevalent among tobacco users (27.8%) than non-users (21.1%). However, the prevalence of hypertension is more among women tobacco users (30.7%) than men (26.8%). The hypertension prevalence is also higher in alcohol drinkers (30.8%) than non-drinkers (21.7%).

The gap between men’s and women’s tobacco and alcohol use is not unusual. It aligns with the Global Adult Tobacco Survey (2016–2017) 19% of men and 2% of women smoke tobacco in India [21]. It may be pointed out that traditionally, tobacco and alcohol usage is significantly higher in men than in women in the Indian sub-continent. This could be attributed to cultural disapproval prohibiting women from smoking and drinking in India. Under-reporting of tobacco and alcohol use by women is also partly responsible.

Hypertension is found to be more prevalent among obese people, BMI greater than or equal to 30 kg/m^2^ (28.8%) and less in thin persons, BMI of less than 18.5 kg/m^2^ (6.1%). Also, a higher hypertension prevalence was observed among those whose BMI was not measured (30.4%).

Hypertension prevalence is high among the person who has an increased risk of metabolic complications (18.4%) when measuring waist circumference. It is higher among men (38.5%) with a waist circumference above 94 cm and women with above 80 cm waist circumference (17.6%).

The prevalence of hypertension is found to be high (40.2%) among those who have a random blood glucose level above 140 mg/dl. A similar pattern of hypertension prevalence is found for both men (40.3%) and women (40.1%) among those who have a high random blood glucose level.

## Prevalence of hypertension across states in India

The finding indicates that the prevalence of hypertension in India is 22.6%. There are 22 states or union territories (UTs) above the national prevalence of hypertension, and only 14 states or UTs are less than the national average. (Table 2)

**Table 2:** Prevalence of hypertension among men and women age 15 and over, according to state/union territory, India, 2019-21

		Men		Women		Overall	
**State code**	**State Name**	Prevalence of hypertension (%)	Weighted sample	Prevalence of hypertension (%)	Weighted sample	Prevalence of hypertension (%)	Weighted sample
-	**India**	**24.1**	**7**,**85**,**611**	**21.2**	**9**,**22**,**631**	**22.6**	**17**,**08**,**241**
1	Jammu & Kashmir	21.8	4,221	22.3	5,334	22.1	9,555
2	Himachal Pradesh	24.4	4,786	22.2	5,820	23.2	10,606
3	Punjab	37.9	17,093	31.1	19,628	34.2	36,721
4	Chandigarh	29.8	418	25.2	579	27.1	997
5	Uttarakhand	32.3	5,307	23.0	7,338	26.9	12,645
6	Haryana	25.3	15,976	21.0	18,133	23.0	34,109
7	NCT of Delhi	32.8	10,580	24.1	12,686	28.0	23,266
8	Rajasthan	17.9	49,200	15.3	57,232	16.5	1,06,431
9	Uttar Pradesh	21.8	1,20,851	18.3	1,47,465	19.9	2,68,317
10	Bihar	18.4	59,480	15.9	80,176	17.0	1,39,657
11	Sikkim	41.4	336	34.6	374	37.9	710
12	Arunachal Pradesh	33.2	683	24.9	730	28.9	1,413
13	Nagaland	29.5	682	23.7	730	26.5	1,412
14	Manipur	33.6	1,469	23.2	1,719	28.0	3,187
15	Mizoram	25.0	768	17.8	806	21.3	1,574
16	Tripura	23.1	2,731	21.1	2,972	22.0	5,702
17	Meghalaya	21.8	1,635	18.9	1,881	20.3	3,516
18	Assam	21.2	18,488	19.7	20,849	20.4	39,337
19	West Bengal	20.1	58,123	20.2	66,542	20.2	1,24,665
20	Jharkhand	22.4	20,732	17.7	24,540	19.9	45,272
21	Odisha	25.6	27,723	22.3	33,843	23.8	61,566
22	Chhattisgarh	27.6	21,432	23.5	23,156	25.5	44,589
23	Madhya Pradesh	22.5	48,164	20.4	53,621	21.4	1,01,785
24	Gujarat	20.1	45,240	20.3	46,424	20.2	91,664
25	Dadra & Nagar Haveli and Daman & Diu	17.2	275	16.4	262	16.8	537
27	Maharashtra	24.1	79,139	22.7	86,757	23.4	1,65,896
28	Andhra Pradesh	29.2	33,584	25.4	38,992	27.2	72,576
29	Karnataka	28.6	38,901	26.0	45,642	27.2	84,542
30	Goa	35.9	454	32.0	655	33.6	1,110
31	Lakshadweep	24.9	49	24.7	64	24.8	113
32	Kerala	32.3	21,898	30.1	28,555	31.1	50,452
33	Tamil Nadu	30.1	54,849	24.8	64,821	27.2	1,19,670
34	Puducherry	30.1	849	22.8	1,021	26.1	1,870
35	Andaman & Nicobar Islands	29.7	275	24.9	279	27.3	554
36	Telangana	31.5	19,127	26.1	22,894	28.6	42,021
37	Ladakh	19.9	93	17.5	109	18.6	202

**Fig. 1:**
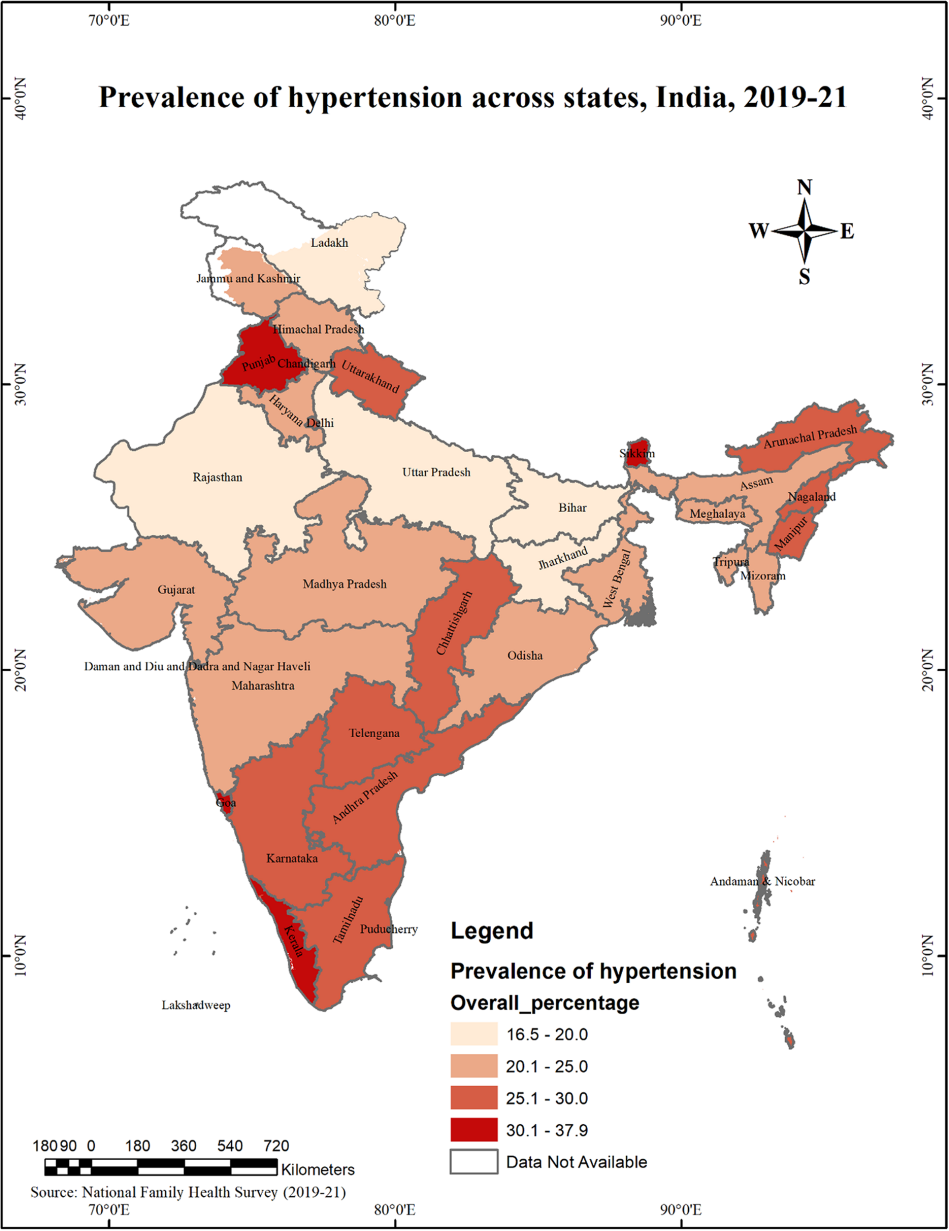
The overall prevalence of hypertension across states in India, 2019-21

The prevalence of hypertension is found to be highest in Sikkim (37.9%), followed by Punjab (34.2%), Goa (33.6%), Kerala (31.1%), Arunachal Pradesh (28.9%), Telangana (28.6%), NCT of Delhi (28.0%), Manipur (28.0%), Andaman & Nicobar Islands (27.3%), Karnataka (27.2%). (Fig. 1)

On the other hand, the proportion of the population suffering from hypertension is relatively low in states such as Rajasthan (16.5%), followed by Dadra & Nagar Haveli and Daman & Diu (16.8%), Bihar (17%), Ladakh (18.6%), Uttar Pradesh (19.9%), Jharkhand (19.9%), West Bengal (20.2%), Gujarat (20.2%), Meghalaya (20.3%), Assam (20.4%). (Fig. 1)

**Fig. 2:**
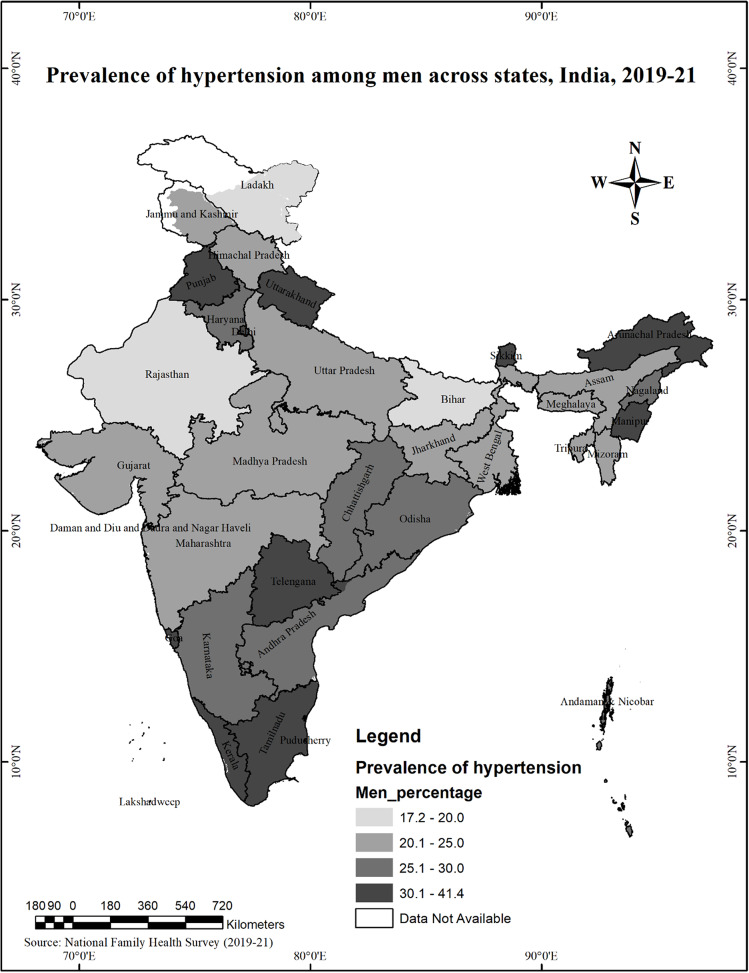
Prevalence of Hypertension among men across states in India, 2019-21

**Fig. 3:**
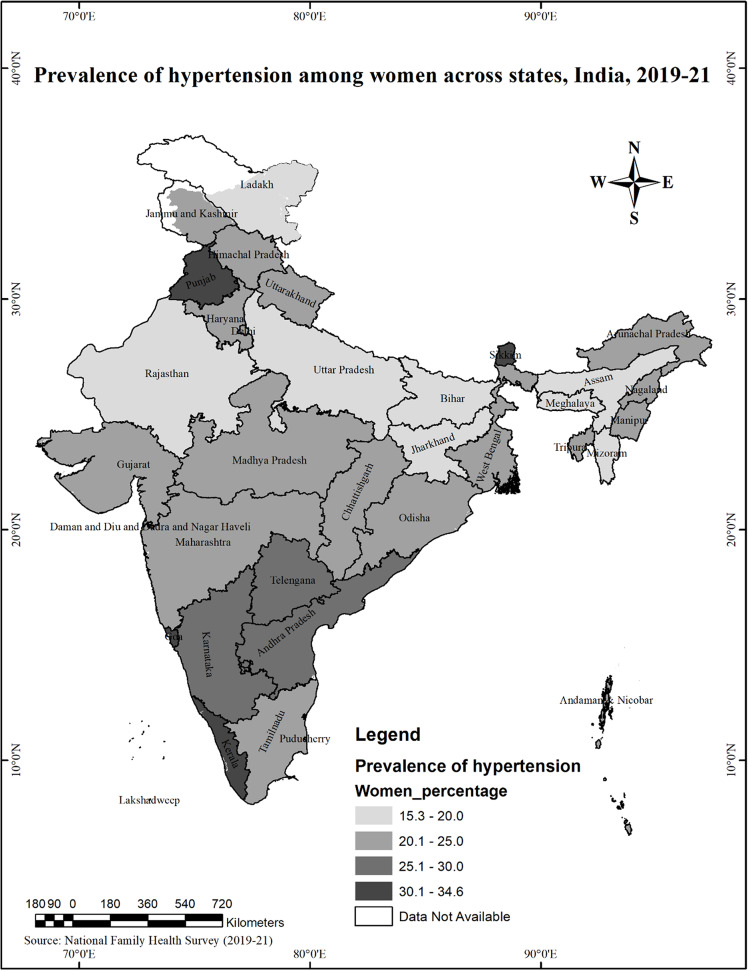
Prevalence of Hypertension among women across states in India, 2019-21

The findings highlighted that the prevalence of hypertension is higher in men than women in most states and UTs, except in West Bengal, Gujarat and Jammu and Kashmir. The gender difference in the prevalence of hypertension is highest in Manipur (Men 33.6%, Women 23.2%), followed by Uttarakhand (Men 32.3%, Women 23%), NCT of Delhi (Men 32.8%, Women 24.1%), Arunachal Pradesh (Men 33.2%, Women 24.9%), Puducherry (Men 30.1%, Women 22.8%). *(*Figs. 2 and 3*)*

### Predictors of hypertension from background characteristics

Table 3 reveals that, after adjusting the sociodemographic characteristics and behavioural risk factors, the predictors of hypertension among the background characteristics for men, women and overall persons. It has been statistically evident that the independent variable such as increasing age (a person of age 60 years or over is ten times more [OR: 8.97 (CI: 8.83–9.12)] likely to have hypertension compared to a person of age 15 to 29 years). The same pattern of the likelihood of hypertension is found in both men [OR: 7.41 (CI: 7.25–7.58)] and women [OR: 8.83 (CI: 8.57–9.10)]. However, women aged 60 or over are more likely hypertensive than men of the same age.

**Table 3:** Binary logistics regression for the association of hypertension and sociodemographic characteristics and behavioural risk factors, India, 2019-21

Background characteristics	Men		Women		Overall	
	Crude OR (95% CI)	Adjusted OR (95% CI)	Crude OR (95% CI)	Adjusted OR (95% CI)	Crude OR (95% CI)	Adjusted OR (95% CI)
**Age of Household Member**						
15–29^®^	1	1	1	1	1	1
30–44	3.04*** (2.99–3.09)	2.49*** (2.43–2.54)	3.61*** (3.54–3.68)	2.77*** (2.72–2.83)	3.27*** (3.23–3.31)	2.85*** (2.8–2.89)
45–59	5.74*** (5.65–5.84)	4.48*** (4.38–4.58)	10.23*** (10.05–10.43)	5.42*** (5.29–5.56)	7.62*** (7.52–7.71)	5.54*** (5.45–5.62)
60 and over	9.20*** (9.04–9.36)	7.41*** (7.25–7.58)	20.82*** (20.42–21.22)	8.83*** (8.57–9.10)	13.77*** (13.59–13.94)	8.97*** (8.83–9.12)
**Type of Place of Residence**						
Urban^®^	1	1	1	1	1	1
Rural	0.82*** (0.81–0.83)	0.96*** (0.94–0.97)	0.83*** (0.82–0.84)	0.94*** (0.93–0.96)	0.82*** (0.82–0.83)	0.95*** (0.94–0.96)
**Region of Residence**						
North^®^	1	1	1	1	1	1
Central	0.89*** (0.87–0.90)	1.03* (1.01–1.05)	0.93*** (0.91–0.94)	1.09*** (1.06–1.11)	0.91*** (0.9–0.92)	1.05*** (1.04–1.07)
East	0.79*** (0.78–0.81)	0.84*** (0.82–0.85)	0.88*** (0.87–0.9)	0.98* (0.96-1.00)	0.84*** (0.83–0.85)	0.91*** (0.89–0.92)
Northeast	0.91*** (0.88–0.94)	0.98 (0.95–1.01)	0.99 (0.96–1.02)	1.14*** (1.10–1.18)	0.95*** (0.93–0.97)	1.06*** (1.03–1.09)
West	0.89*** (0.87–0.91)	0.88*** (0.86–0.90)	1.08*** (1.06–1.11)	1.02* (1.00-1.05)	0.99** (0.97-1.00)	0.95*** (0.94–0.97)
South	1.30*** (1.28–1.32)	1.14*** (1.12–1.17)	1.36*** (1.34–1.39)	1.17*** (1.15–1.20)	1.33*** (1.31–1.35)	1.16*** (1.14–1.17)
**Religion of The Household Head**						
Hindu^®^	1	1	1	1	1	1
Muslim	0.87*** (0.86–0.89)	0.97** (0.96–0.99)	1.00 (0.98–1.01)	1.10*** (1.08–1.12)	0.94*** (0.92–0.95)	1.04*** (1.02–1.05)
Others	1.38*** (1.35–1.41)	1.20*** (1.18–1.23)	1.37*** (1.34–1.40)	1.18*** (1.15–1.21)	1.38*** (1.36–1.40)	1.18*** (1.16–1.20)
**Caste/Tribe of The Household Head**						
None of them^®^	1	1	1	1	1	1
Schedule Caste (SC)	0.83*** (0.82–0.85)	0.97*** (0.95–0.99)	0.77*** (0.76–0.78)	0.91*** (0.89–0.92)	0.80*** (0.79–0.81)	0.94*** (0.93–0.95)
Schedule Tribe (ST)	0.81*** (0.79–0.82)	1.04*** (1.02–1.07)	0.78*** (0.77–0.80)	1.05*** (1.02–1.07)	0.80*** (0.78–0.81)	1.04*** (1.03–1.06)
Other Backward Classes (OBC)	0.88*** (0.87–0.89)	0.92*** (0.91–0.93)	0.85*** (0.84–0.86)	0.89*** (0.88–0.91)	0.87*** (0.86–0.87)	0.91*** (0.9–0.92)
**Current Marital Status**						
Married^®^	1	1	1	1	1	1
Others	0.35*** (0.34–0.35)	0.85*** (0.83–0.86)	1.06*** (1.05–1.08)	1.10*** (1.09–1.12)	0.65*** (0.64–0.65)	1.06*** (1.05–1.07)
**Highest Educational Level Attained**						
Non-literate^®^	1	1	1	1	1	1
Primary	0.97*** (0.95–0.99)	1.13*** (1.11–1.16)	0.80*** (0.79–0.81)	1.10*** (1.08–1.12)	0.86*** (0.85–0.87)	1.05*** (1.04–1.07)
Secondary	0.68*** (0.67–0.69)	1.15*** (1.13–1.17)	0.38*** (0.38–0.39)	0.94*** (0.92–0.95)	0.52*** (0.51–0.52)	0.99* (0.98-1.00)
Higher	0.68*** (0.67–0.69)	1.16*** (1.13–1.19)	0.28*** (0.27–0.28)	0.72*** (0.7–0.74)	0.47*** (0.46–0.47)	0.92*** (0.9–0.93)
**Wealth Index**						
Poorest^®^	1	1	1	1	1	1
Poorer	1.08*** (1.06–1.10)	1.08*** (1.06–1.1)	1.04*** (1.02–1.06)	1.04*** (1.02–1.06)	1.06*** (1.05–1.07)	1.07*** (1.05–1.08)
Middle	1.24*** (1.22–1.27)	1.19*** (1.17–1.22)	1.18*** (1.16–1.19)	1.13*** (1.11–1.15)	1.21*** (1.19–1.22)	1.18*** (1.16–1.19)
Richer	1.46*** (1.43–1.48)	1.33*** (1.3–1.36)	1.28*** (1.26–1.3)	1.18*** (1.15–1.2)	1.36*** (1.35–1.38)	1.27*** (1.25–1.29)
Richest	1.73*** (1.70–1.76)	1.43*** (1.4–1.47)	1.45*** (1.43–1.47)	1.23*** (1.2–1.26)	1.58*** (1.56–1.60)	1.34*** (1.32–1.37)
**Smokes or Uses Tobacco**						
No^®^	1	1	1	1	1	1
Yes	1.27*** (1.25–1.28)	0.92*** (0.90–0.93)	1.74*** (1.72–1.77)	0.99 (0.97–1.01)	1.43*** (1.42–1.44)	0.91*** (0.90–0.92)
**Drinks Alcohol**						
No^®^	1	1	1	1	1	1
Yes	1.48*** (1.46–1.49)	1.28*** (1.26–1.3)	1.66*** (1.59–1.72)	1.17*** (1.12–1.22)	1.56*** (1.54–1.58)	1.26*** (1.24–1.28)
**Body Mass Index (BMI in kg/m2)**						
Normal (BMI 18.5–24.9) ^®^	1	1	1	1	1	1
Thin (BMI < 18.5)	0.46*** (0.43–0.49)	0.67*** (0.62–0.72)	0.60*** (0.58–0.61)	0.86*** (0.84–0.89)	0.57*** (0.55–0.58)	0.80*** (0.78–0.82)
Overweight (BMI 25.0-29.9)	2.29*** (2.20–2.39)	1.46*** (1.39–1.53)	2.28*** (2.24–2.33)	1.49*** (1.46–1.52)	2.28*** (2.24–2.32)	1.55*** (1.52–1.58)
Obese (BMI ≥ 30.0)	3.44*** (3.20–3.69)	1.83*** (1.68–1.99)	3.72*** (3.63–3.81)	2.13*** (2.07–2.19)	3.55*** (3.47–3.63)	2.12*** (2.07–2.18)
BMI not measured	1.75*** (1.71–1.79)	1.68** (1.18–2.39)	7.83*** (7.73–7.94)	1.80*** (1.57–2.06)	3.82*** (3.78–3.86)	1.82*** (1.61–2.06)
**Waist Circumference**						
Normal (women: (≤ 80 cm); men (≤ 94 cm)) ^®^	1	1	1	1	1	1
Increased risk of metabolic complications (women: (> 80 cm); men (> 94 cm))	2.59*** (2.55–2.63)	1.51*** (1.43–1.60)	3.39*** (3.25–3.54)	1.34*** (1.31–1.36)	2.30*** (2.27–2.33)	1.16*** (1.14–1.18)
Not measured	9.90*** (9.76–10.04)	0.69* (0.49–0.98)	1.79*** (1.76–1.83)	1.19** (1.04–1.37)	4.45*** (4.40–4.50)	0.98 (0.86–1.11)
**Random Blood Glucose Level**						
Normal (≤ 140 mg/dl) ^®^	1	1	1	1	1	1
High (> 140 mg/dl)	3.22*** (3.18–3.27)	1.67*** (1.65–1.70)	2.70*** (2.67–2.74)	1.61*** (1.59–1.64)	2.97*** (2.94-3.00)	1.65*** (1.64–1.67)
Not measured	10.90*** (10.59–11.23)	10.93*** (10.51–11.36)	14.53*** (14-15.07)	7.56*** (7.31–7.81)	12.16*** (11.89–12.44)	8.92*** (8.70–9.15)
***Sample size (n)***	*7*,*85*,*611*		*9*,*22*,*630*		*17*,*08*,*241*	

*Note*^®^ - Reference category, OR - Odds Ratio, CI - Confidence Interval, *** *p* < 0.01, ** *p* < 0.05, * *p* < 0.10

A person residing in a rural area is slightly less likely to have hypertension than one in an urban area [OR: 0.95 (CI: 0.94–0.96)]. Almost the same observation has been found in both men [OR: 0.96 (CI: 0.94–0.97)] and women [OR: 0.94 (CI: 0.93–0.96)].

A statistically significant finding observes that, when compared to the Northern person, the person residing in Central [OR: 1.05 (CI: 1.04–1.07)], North-eastern [OR: 1.06 (CI: 1.03–1.09)], or Southern [OR: 1.16 (CI: 1.14–1.17)] region are more likely to have hypertension, and the person residing in Eastern [OR: 0.91 (CI: 0.89–0.92)], or Western [OR: 0.95 (CI: 0.94–0.97)] region have less likely to have hypertension. A similar pattern has been observed for both men and women.

A person belonging to a Muslim [OR: 1.04 (CI: 1.02–1.05)] and a person from other than Hindu or Muslim [OR: 1.18 (CI: 1.16–1.20)] religions are more likely to have hypertension than a person from the Hindu religion. The same observation has been found in men [Others men; OR: 1.20 (CI: 1.18–1.23)] and women [Muslim: OR: 1.10 (CI: 1.08–1.12), Others: OR: 1.18 (CI: 1.15–1.21)].

There is enough evidence to conclude that when comparing the person who does not belong to any caste or tribe, the SCs [OR: 0.94 (CI: 0.93–0.95)] and OBCs [OR: 0.91 (CI: 0.90–0.92)] are less likely to have hypertension. However, STs are slightly more likely [OR: 1.04 (CI: 1.03–1.06)] to have hypertension. The finding is the same for men and women.

It has been significantly observed that men whose marital status is others are less likely [OR: 0.85 (CI: 0.83–0.86)] to have hypertension than currently married men. Nevertheless, women whose marital status is others are more likely [OR: 1.10 (CI: 1.09–1.12)] to have hypertension than currently married women.

Level of education, when comparing non-literates, it has been significantly found that men with a higher level of education have a higher chance [OR: 1.16 (CI: 1.13–1.19)] of having hypertension. Nevertheless, women with a higher level of education have a lesser chance [OR: 0.72 (CI: 0.70–0.74)] of having hypertension.

It has been significant enough to conclude that both men and women of the richest wealth index are more likely [Men: OR: 1.43 (CI: 1.40–1.47) Women: OR 1.23 (CI: 1.20–1.26)] to have hypertension than both of the poorest.

Men and women who consume tobacco are significantly more likely to have hypertension in crude analysis; however, after the influence of other predictors in multivariate analysis, the result shows a negative association. Although, men [OR: 1.28 (CI: 1.26–1.30)] and women [OR: 1.17 (CI: 1.12–1.22)] who drink alcohol are significantly more likely to have hypertension than non-drinkers.

There is statistically enough evidence to conclude that, when compared with persons with a normal BMI, the persons whose BMI is Thin are less likely [OR: 0.80 (CI: 0.78–0.82)] to have hypertension both in men [OR: 0.67 (CI: 0.62–0.72)] and women [OR: 0.86 (CI: 0.84–0.89)]. Overweight [OR: 1.55 (CI: 1.52–1.58)] and Obese [OR: 2.12 (CI: 2.07–2.18)] persons are more likely to have hypertension. However, obese women have a higher chance of hypertension than obese men when compared to persons with normal BMI of both men and women separately. Men and women with a BMI of Overweight, Obese and whose BMI is not measured are more likely to have hypertension.

A statistically significant association is found with the waist circumference; the persons with an increased risk of metabolic complications are more likely [OR: 1.16 (CI: 1.14–1.18)] to have hypertension than those measured as normal. Men and women who have an increased risk of metabolic complications (more than 94 cm and 80 cm of waist circumference among men and women, respectively) are more likely [Men: OR: 1.51 (CI: 1.43–1.60) Women: OR 1.34 (CI: 1.31–1.36)] to have hypertension when compared to the person who has measured as normal waist circumference.

Random blood glucose level is found to be a statistically significant risk factor for hypertension. A person who has a high (more than 140 mg/dl) blood glucose level is more likely [OR: 1.65 (CI: 1.64–1.67)] to have hypertension compared to a person with a normal random blood glucose level. A similar association is found for both men [OR: 1.67 (CI: 1.65–1.70)] and women [OR: 1.61 (CI: 1.59–1.64)].

## Discussion

This study provides estimates of the prevalence of hypertension across states in India. It examines socioeconomic and risk factors associated with this condition, by exploring the latest data from the fifth round (2019–21) of NFHS. The hypertension prevalence is found to be 22.6%. It is more prevalent in men (24.1%) than women (21.2%), which is supported by the WHO estimate as the overall prevalence of hypertension in India was 23.5% and gender-specific prevalence was 24.2% and 22.7% among men and women, respectively [22].

Men have a higher prevalence of hypertension than women. Similarly, various studies came out with a higher percentage of hypertension in men than women [23–25]. One of the possible explanations for this gender disparity in hypertension prevalence could be partially due to biological sex differences and partially due to behavioural risk factors like smoking, alcohol consumption, or physical activity. Other than that, women are more interested in health care services utilization and also more frequently report their poor health; therefore, they are more likely to have better health [26, 27].

Age is found to be an important factor for hypertension. As age increases, so does the prevalence of hypertension among both genders. Similar findings were reported by a few other studies also, where advancing age is positively related to hypertension [5, 24, 27–31]. The aorta and arteries walls will be stiffened with increasing age, and this contributes to the high prevalence of hypertension in older age groups [3].

At the national level, the prevalence of hypertension is found to be higher in urban areas in comparison with rural areas. Although the prevalence of hypertension is higher in urban areas compared to rural areas, the difference is narrowing. It indicates that hypertension is spreading rapidly, even in rural populations. Studies found that the primary healthcare infrastructure in rural areas predominantly addresses infectious diseases and reproductive and child health, particularly in India, leaving limited resources for the management of hypertension. Consequently, individuals may need to seek assistance from private healthcare providers to address hypertension and its related conditions, potentially increasing their financial burdens [25, 30]. Urbanisation also causes changes in dietary habits and decreased physical activity, leading to obesity and, ultimately, hypertension [3].

The finding of relatively lower hypertension prevalence in poorer states is consistent with evidence from the latest burden of disease study that classified these states as having low epidemiological transition level [32].

In the present study, marital status, education, wealth index, alcohol use, BMI, waist circumference and random blood glucose level are significantly associated with hypertension. Literacy level and being too rich are associated with hypertension. The higher education level is found to be correlated to hypertension, contrary to men and women in the present study. Highly educated men are more likely to be hypertensive, whereas highly educated women are less likely to be hypertensive. There are a few studies also supported this finding [27, 33]. It could be because higher education imparts better knowledge and information about hypertension; subsequently, those people with higher education had a healthier lifestyle.

According to a WHO report, cigarette use was the second leading cause of death worldwide, and alcohol consumption was the third leading risk factor in developed countries [34]. This study indicated the positive association between alcohol use and hypertension. Hypertension is found to be more prevalent in alcohol users, both in men and women, as compared to non-users. Other studies support this finding [23, 29, 31, 35, 36]. Surprisingly, the use of tobacco is found to correlate with the risk of hypertension negatively. While it is difficult to explain, according to a study that examined the life-course impact of smoking on hypertension, no statistically significant relationship was found between smoking and the risk of hypertension in the age group younger than 35, though smoking was found to be significantly associated with hypertension in the later ages [37].

This analysis revealed that being overweight or obese, as measured by BMI, was a significant modifiable risk factor for developing hypertension. In comparison to normal subjects, overweight subjects had a twofold risk of hypertension, and obese subjects had a nearly threefold risk. According to previous studies, there is a strong correlation between increasing BMI and rising rates of hypertension [3, 5, 14, 23, 25, 27–29, 31, 38]. One possible reason for the positive relationship between obesity and hypertension is that increased weight increases heart rate and vascular resistance of blood vessels [3].

It has been observed that a person with an increased risk of metabolic complications (waist circumference higher than 94 cm for men and 80 for women) has a potentially higher risk of having hypertension. A similar finding was also observed in a few studies; it has been found that there was a significant association with approximately twice the risk of hypertension [14, 15, 25, 38]. This result aligns with other research showing that central obesity, as indicated by waist circumference, is a strong predictor of high blood pressure. For instance, a study explains that excess abdominal fat can lead to stiffer arteries and insulin resistance, both of which can raise blood pressure [39].

The random blood glucose level finding revealed that the person with a high blood glucose level has a high risk for hypertension. The association has been observed according to the studies [3, 5, 15, 24, 37]. Elevated blood glucose levels can cause hypertension through several mechanisms, including damage to the blood vessels and increased pressure on the heart, as discussed in previous studies. In a study, it has been highlighted how the dual burden of diabetes and hypertension is becoming a serious public health challenge in India [40]. Supporting this, the Chennai Urban Rural Epidemiology Study (CURES) reported that individuals with impaired glucose tolerance or diabetes have a significantly higher prevalence of hypertension [38]. This suggests that the combination of obesity, high glucose levels, and hypertension, often referred to as metabolic syndrome, is increasingly common in India and poses a significant public health risk.

### Limitations

The study has a few limitations. A cross-sectional design limits the ability to establish causality between the identified risk factors and hypertension, allowing only for associations to be determined. Potential confounders, despite adjustments, may still influence observed associations. Self-reported data on behaviours such as tobacco use and alcohol consumption may introduce reporting bias, particularly underreporting of tobacco use and alcoholism among women, also in socially sensitive behaviours, affecting the accuracy of the associations observed with hypertension. The tobacco variable was highly influenced by the “age” variable in the multivariable analysis, which resulted in a negative association in the multivariable analysis. Blood pressure measurements were obtained for a large sample. Still, height and weight data were collected for a smaller subset, which may introduce bias when examining the relationship between BMI, waist circumference and hypertension. These limitations highlight the need for cautious interpretation of the results and underscore areas for further longitudinal studies.

## Conclusion

The prevalence of hypertension is rapidly increasing in India. Currently, one in every fifth of individuals is hypertensive in India, and nearly 50% of individuals aged 60 years and above have it. Age is the strongest non-modifiable predictor of hypertension for both men and women. However, ageing women have higher odds of hypertension than ageing men, and this distinction becomes much clearer after their reproductive age (49 years or above). Furthermore, it is more prevalent in the Southern part of the country. Hypertension is higher among those who reside in urban areas, who are elderly, who belong to a no-caste or other than Hindus and Muslim religion, who are not currently married, who are non-literate, who are in the richest wealth index, who consume alcohol, who are overweight or obese, who are at an increased risk of metabolic complications in waist circumference measurement, and who have a high random blood glucose level. Women with a higher education level have less chance of having hypertension than men, indicating that women’s education is essential for good health. There is various background and risk factors have been found to be responsible for an increase in hypertension. Obese women, based on BMI, have higher odds of hypertension than men. However, hypertension prevalence is slightly higher among men who are overweight or obese compared to women. The positive association of hypertension with alcohol use, overweight, obesity, increased waist circumference, and high blood glucose levels emphasize the need for comprehensive public health interventions that address these modifiable risk factors. The policymakers must take this on a large scale, in both urban and rural areas, as the rural-urban differences were small, implying that hypertension is spreading very fast, even in the rural population. Existing interventions should improve for better management of hypertension. Policy measures should be taken to improve the hazardous growing social pressures of survival responsible for lifestyle changes such as healthier diets and increased physical activity, along with regular monitoring of these modifiable risk factors for both men and women, which could have a significant influence on reducing the growing burden of hypertension in India.

## Electronic supplementary material

Below is the link to the electronic supplementary material.

Supplementary Material 1

## Data Availability

NFHS data is a nationally representative data set that is available freely in the public domain. For more details, visit www.measuredhs.com. It can be accessed after the request is made to and approved by the DHS program.

## Abbreviations

BMI: Body Mass Index
CURES: Chennai Urban Rural Epidemiology Study
CI: Confidence Interval
DBP: Diastolic Blood Pressure
DHS: Demographic and Health Survey
ICF: Inner City Fund
ITDA: Integrated Tribal Development Authority
NCT: National Capital Territory
NFHS: National Family Health Survey
NNMB: National Nutrition Monitoring Bureau
OBC: Other Backward Classes
OR: Odds Ratio
SBP: Systolic Blood Pressure
SC: Scheduled Caste
ST: Scheduled Tribe
UTs: Union Territories
WHO: World Health Organization

## References

[1] NCD. Non communicable diseases [Internet]. 2022 [cited 2023 Mar 6]. https://www.who.int/news-room/fact-sheets/detail/noncommunicable-diseases

[2] GBD. Institute for Health Metrics and Evaluation. 2019 [cited 2023 Mar 6]. Global Burden of Disease 2019 (GBD 2019). https://vizhub.healthdata.org/gbd-results/

[3] Abebe SM, Berhane Y, Worku A, Getachew A. Prevalence and Associated factors of hypertension: a Crossectional Community based study in Northwest Ethiopia. PLoS ONE. 2015;10(4):e0125210. 25909382 10.1371/journal.pone.0125210 PMC4409323

[4] Ahmed A, Rahman M, Hasan R, Shima SA, Faruquee MH, Islam T, et al. Hypertension and associated risk factors in some selected rural areas of Bangladesh. Int J Res Med Sci. 2014;2(3):925–31.

[5] Erem C, Hacihasanoglu A, Kocak M, Deger O, Topbas M. Prevalence of prehypertension and hypertension and associated risk factors among Turkish adults: Trabzon Hypertension Study. J Public Health Oxf Engl. 2009;31(1):47–58. 10.1093/pubmed/fdn078 18829520

[6] WHO. Hypertension [Internet]. 2021 [cited 2023 Jan 5]. https://www.who.int/news-room/fact-sheets/detail/hypertension

[7] Chobanian AV, Bakris GL, Black HR, Cushman WC, Green LA, Izzo JL, et al. Seventh report of the Joint National Committee on Prevention, detection, evaluation, and treatment of high blood pressure. Hypertens Dallas Tex 1979. 2003;42(6):1206–52. 10.1161/01.HYP.0000107251.49515.c2 14656957

[8] Al-Makki A, DiPette D, Whelton PK, Murad MH, Mustafa RA, Acharya S, et al. Hypertension pharmacological treatment in adults: a World Health Organization Guideline Executive Summary. Hypertension. 2022;79(1):293–301. 34775787 10.1161/HYPERTENSIONAHA.121.18192 PMC8654104

[9] Prabakaran J, Vijayalakshmi N, VenkataRao E. Prevalence of hypertension among urban adult population (25–64 years) of Nellore, India. Int J Res Dev Health. 2013;1:42–9.

[10] WHO. Global Hearts Initiative [Internet]. 2016 [cited 2023 Feb 5]. https://www.who.int/news/item/15-09-2016-global-hearts-initiative

[11] WHO. Improving hypertension control in 3 million people: country experiences of programme development and implementation [Internet]. 2020 [cited 2023 Feb 15]. https://www.who.int/publications-detail-redirect/improving-hypertension-control-in-3-million-people-country-experiences-of-programme-development-and-implementation

[12] World Health Assembly 66. Draft comprehensive global monitoring framework and targets for the prevention and control of noncommunicable diseases: formal meeting of the Member States to conclude the work on the comprehensive global monitoring framework, including indicators, and a set of voluntary global targets for the prevention and control of noncommunicable diseases: report by the Director-General [Internet]. World Health Organization. 2013 [cited 2023 Mar 5]. Report No.: A66/8. https://apps.who.int/iris/handle/10665/105633

[13] Laxmaiah A, Meshram II, Arlappa N, Balakrishna N, Rao KM, Reddy CG, et al. Socio-economic & demographic determinants of hypertension & knowledge, practices & risk behaviour of tribals in India. Indian J Med Res. 2015;141(5):697–708. 26139790 10.4103/0971-5916.159592 PMC4510771

[14] Ragavan RS, Ismail J, Evans RG, Srikanth VK, Kaye M, Joshi R, et al. Combining general and central measures of adiposity to identify risk of hypertension: a cross-sectional survey in rural India. Obes Res Clin Pract. 2023;17(3):249–56. 37142499 10.1016/j.orcp.2023.04.005

[15] Indrapal M, Nagalla B, Varanasi B, Rachakulla H, Avula L. Socio-demographic factors, overweight/obesity and nutrients associated with hypertension among rural adults (≥ 18 years): findings from National Nutrition Monitoring Bureau survey. Indian Heart J. 2022;74(5):382–90. 36055373 10.1016/j.ihj.2022.08.006 PMC9647655

[16] Ghosh S, Kumar M. Prevalence and associated risk factors of hypertension among persons aged 15–49 in India: a cross-sectional study. BMJ Open. 2019;9(12):e029714. 31848161 10.1136/bmjopen-2019-029714 PMC6937064

[17] Anchala R, Kannuri NK, Pant H, Khan H, Franco OH, Di Angelantonio E, et al. Hypertension in India: a systematic review and meta-analysis of prevalence, awareness, and control of hypertension. J Hypertens. 2014;32(6):1170. 24621804 10.1097/HJH.0000000000000146 PMC4011565

[18] collaborators NCDC2030. NCD countdown 2030: worldwide trends in non-communicable disease mortality and progress towards sustainable development goal target 3.4. Lancet Lond Engl. 2018;392(10152):1072–88. 10.1016/S0140-6736(18)31992-5 30264707

[19] IIPS ICF. National Family Health Survey (NFHS-5), 2019-21: India [Internet]. Mumbai: International Institute for Population Sciences (IIPS) and ICF; 2021 [cited 2023 Feb 5]. https://iipsindia.ac.in/content/national-family-health-survey-nfhs-5-india-report

[20] Pickering TG, Hall JE, Appel LJ, Falkner BE, Graves JW, Hill MN, et al. Recommendations for blood pressure measurement in humans: an AHA scientific statement from the Council on High Blood Pressure Research Professional and Public Education Subcommittee. J Clin Hypertens Greenwich Conn. 2005;7(2):102–9. 10.1111/j.1524-6175.2005.04377.x PMC8109470 15722655

[21] GYTS. Global Adult Tobacco Survey GATS 2 India 2016–17 [Internet]. Tata Institute of Social Sciences (TISS) and Ministry of Health and Family Welfare. 2017. https://ntcp.mohfw.gov.in/assets/document/surveys-reports-publications/Global-Adult-Tobacco-Survey-Second-Round-India-2016-2017.pdf

[22] WHO. WHO, World Health O. 2015 [cited 2023 Mar 1]. Global Health Observatory data repository 2015. https://apps.who.int/gho/data/view.main.NCDBPCREGv?lang=en

[23] Dhungana RR, Pandey AR, Bista B, Joshi S, Devkota S. Prevalence and Associated factors of hypertension: a community-based cross-sectional study in municipalities of Kathmandu, Nepal. Int J Hypertens. 2016;2016:e1656938. 10.1155/2016/1656938 PMC4880705 27293880

[24] Gao Y, Chen G, Tian H, Lin L, Lu J, Weng J, et al. Prevalence of hypertension in China: a cross-sectional study. PLoS ONE. 2013;8(6):e65938. 23776574 10.1371/journal.pone.0065938 PMC3679057

[25] Singh R, Sinha RK, Mani C, Singh R, Pal R. Burden and vulnerability of hypertension in a rural population of Patna, Bihar, India. South East Asia J Public Health. 2011;1(1):53–8.

[26] Everett B, Zajacova A. Gender differences in hypertension and hypertension awareness among young adults. Biodemography Soc Biol. 2015;61(1):1–17. 25879259 10.1080/19485565.2014.929488 PMC4896734

[27] Tabrizi JS, Sadeghi-Bazargani H, Farahbakhsh M, Nikniaz L, Nikniaz Z. Prevalence and Associated Factors of Prehypertension and Hypertension in Iranian Population: the Lifestyle Promotion Project (LPP). PLoS ONE. 2016;11(10):e0165264. 27783691 10.1371/journal.pone.0165264 PMC5082665

[28] da Costa JSD, Barcellos FC, Sclowitz ML, Sclowitz IKT, Castanheira M, Olinto MTA, et al. Hypertension prevalence and its associated risk factors in adults: a population-based study in Pelotas. Arq Bras Cardiol. 2007;88:59–65. 17364120 10.1590/s0066-782x2007000100010

[29] Reddy SS, Prabhu GR, Tirupati AP. Indian J Community Med. 2005;30(3):84.

[30] Singh S, Shankar R, Singh GP. Prevalence and Associated Risk factors of hypertension: a cross-sectional study in Urban Varanasi. Int J Hypertens. 2017;2017:e5491838. 10.1155/2017/5491838 PMC5733954 29348933

[31] Wamala JF, Karyabakabo Z, Ndungutse D, Guwatudde D. Prevalence factors associated with Hypertension in Rukungiri District, Uganda - A Community-Based Study. Afr Health Sci [Internet]. 2009 [cited 2023 Mar 5];9(3). https://www.ajol.info/index.php/ahs/article/view/48999 PMC2887031 20589143

[32] Dandona L, Dandona R, Kumar GA, Shukla DK, Paul VK, Balakrishnan K, et al. Nations within a nation: variations in epidemiological transition across the States of India, 1990–2016 in the global burden of Disease Study. Lancet. 2017;390(10111):2437–60. 29150201 10.1016/S0140-6736(17)32804-0 PMC5720596

[33] Vimala A, Ranji SA, Jyosna MT, Chandran V, Mathews SR, Pappachan JM. The prevalence, risk factors and awareness of hypertension in an urban population of Kerala (South India). Saudi J Kidney Dis Transpl. 2009;20(4):685. 19587522

[34] WHO. WHO STEPS surveillance manual: the WHO STEPwise approach to chronic disease risk factor surveillance [Internet]. 2005 [cited 2023 Mar 5]. https://apps.who.int/iris/bitstream/handle/10665/43376/?sequence=1

[35] Kishore J, Gupta N, Kohli C, Kumar N. Prevalence of hypertension and determination of its risk factors in Rural Delhi. Int J Hypertens. 2016;2016:e7962595. 10.1155/2016/7962595 PMC4834167 27127646

[36] Maniyara K, Kodali PB, Thankappan KR. Prevalence, awareness, treatment, control and correlates of prevalence and control of hypertension among older adults in Kerala: a mixed methods study. Indian Heart J. 2023;75(3):185–9. 36963664 10.1016/j.ihj.2023.03.004 PMC10258381

[37] Devi P, Rao M, Sigamani A, Faruqui A, Jose M, Gupta R, et al. Prevalence, risk factors and awareness of hypertension in India: a systematic review. J Hum Hypertens. 2013;27(5):281–7. 22971751 10.1038/jhh.2012.33

[38] Mohan V, Anjana RM, Unnikrishnan R, Venkatesan U, Uma Sankari G, Rahulashankiruthiyayan T, et al. Incidence of hypertension among Asian indians: 10 year follow up of the Chennai Urban Rural Epidemiology Study (CURES-153). J Diabetes Complications. 2020;34(10):107652. 32595016 10.1016/j.jdiacomp.2020.107652

[39] Hall JE, do Carmo JM, da Silva AA, Wang Z, Hall ME. Obesity, kidney dysfunction and hypertension: mechanistic links. Nat Rev Nephrol. 2019;15(6):367–85. 31015582 10.1038/s41581-019-0145-4 PMC7278043

[40] Mohan V, Seedat YK, Pradeepa R. The rising burden of diabetes and hypertension in southeast Asian and African regions: need for effective strategies for prevention and control in primary health care settings. Int J Hypertens. 2013;2013:409083. 23573413 10.1155/2013/409083 PMC3612479

