# Supplementary material for: Hypertension in India: a gender-based study of prevalence and associated risk factors
Source: BMC Public Health. 2024 Oct 1;24:2681. doi: 10.1186/s12889-024-20097-5 (PMC11443669; doi:10.1186/s12889-024-20097-5)
Supplement: Supplementary file 1 — Supplementary Material 1 [file 12889_2024_20097_MOESM1_ESM.docx]

| **Table 1a:** Description of the Sample distribution according to background characteristics | | |
| --- | --- | --- |
| **Background characteristics** | **Overall weighted sample distribution** | |
|  | Percentage (%) | Frequency (N) |
| **Age of Household Member** |  |  |
| 15-29 | 34.5 | 5,89,534 |
| 30-44 | 27.4 | 4,68,223 |
| 45-59 | 21.6 | 3,68,663 |
| 60 and over | 16.5 | 2,81,598 |
| **Sex of the member** |  |  |
| Male | 46.0 | 7,85,611 |
| Female | 54.0 | 9,22,630 |
| **Type of Place of Residence** |  |  |
| Urban | 31.7 | 5,41,808 |
| Rural | 68.3 | 11,66,433 |
| **Region of Residence** |  |  |
| North | 13.7 | 2,34,533 |
| Central | 24.3 | 4,14,690 |
| East | 21.7 | 3,71,160 |
| Northeast | 3.3 | 56,852 |
| West | 15.2 | 2,59,207 |
| South | 21.8 | 3,71,799 |
| **Religion of The Household Head** |  |  |
| Hindu | 83.0 | 14,17,134 |
| Muslim | 11.6 | 1,98,592 |
| Others | 5.4 | 92,515 |
| **Caste/Tribe of The Household Head** | |  |
| None of them^#^ | 26.0 | 4,43,940 |
| Schedule Caste (SC) | 21.8 | 3,71,732 |
| Schedule Tribe (ST) | 9.5 | 1,62,838 |
| Other Backward Classes (OBC) | 42.7 | 7,29,731 |
| **Current Marital Status** |  |  |
| Married | 69.8 | 11,91,486 |
| Unmarried | 30.2 | 5,16,532 |
| **Highest Educational Level Attained** | |  |
| Non-literate | 25.6 | 4,36,638 |
| Primary | 14.0 | 2,39,472 |
| Secondary | 46.4 | 7,92,889 |
| Higher | 14.0 | 2,38,283 |
| **Wealth Index** |  |  |
| Poorest | 18.4 | 3,14,266 |
| Poorer | 19.8 | 3,37,704 |
| Middle | 20.7 | 3,53,095 |
| Richer | 20.9 | 3,57,329 |
| Richest | 20.2 | 3,45,847 |
| **Smokes or Uses Tobacco** |  |  |
| No | 77.0 | 13,14,981 |
| Yes | 23.0 | 3,92,125 |
| **Drinks Alcohol** |  |  |
| No | 90.4 | 15,42,973 |
| Yes | 9.6 | 1,64,035 |
| **Body Mass Index (BMI in kg/m^2^)** |  |  |
| Normal (BMI 18.5-24.9) | 25.4 | 4,33,976 |
| Thin (BMI <18.5) | 7.9 | 1,35,640 |
| Overweight (BMI 25.0-29.9) | 7.7 | 1,31,851 |
| Obese (BMI ≥30.0) | 2.7 | 44,862 |
| BMI not measured | 56.3 | 9,61,912 |
| **Waist Circumference** |  |  |
| Normal (women: (≤80 cm); men (≤94 cm)) | 27.5 | 4,69,532 |
| Increased risk of metabolic complications (women: (>80 cm); men (>94 cm)) | 16.2 | 2,76,947 |
| Not measured | 56.3 | 9,61,762 |
| **Random Blood Glucose Level** |  |  |
| Normal (≤140 mg/dl) | 84.6 | 14,45,543 |
| High (>140 mg/dl) | 13.1 | 2,23,364 |
| Not measured | 2.3 | 39,334 |
| **Total** | **100.0** | **17,08,241** |
| **Note:** ^#^ Including those who do not belong to any caste/tribe, or have chosen not to respond | | |
